# Assessing the quality and educational applicability of AI-generated anterior segment images in ophthalmology

**DOI:** 10.1038/s41598-025-27020-x

**Published:** 2025-11-28

**Authors:** Yizhou Yang, Lifang Bai, Yuecheng Ren, Xuanqiao Lin

**Affiliations:** 1 Department of Ophthalmology, Eye, Ear, Nose, and Throat Hospital of Fudan University, Shanghai, People’s Republic of China; 2 Department of Ophthalmology, Jiaxing Traditional Chinese Medicine Hospital Affiliated With Zhejiang, Chinese Medical University, Jiaxing, 314001 Zhejiang Province People’s Republic of China

**Keywords:** Medical education, Artificial intelligence, Text-to-image, Ophthalmology training, Anterior segment, Diseases, Health care, Medical research

## Abstract

Text-to-image (T2I) artificial intelligence models are being increasingly explored in medical education, yet their utility in ophthalmology remains unclear. Slit-lamp anterior segment photography, as a cornerstone of ophthalmic training, provides an ideal context for evaluation. We assessed 40 cases of anterior segment disease. The text descriptions were generated using GPT-4o, and the corresponding images were synthesized via Sora Turbo. Readability was analysed with the Flesch Reading Ease (FRE), Flesch‒Kincaid Grade Level (FKGL), and Gunning Fog Scale (GFS). Twenty ophthalmologists (10 juniors, 10 seniors) rated image-text pairs across five dimensions—text accuracy, image reliability, recognizability, educational value, and generation stability—using a 5-point Likert scale. Entities with distinct morphological features, such as cataracts and subconjunctival haemorrhages, received the highest total scores, whereas those with entropion and corneal foreign bodies scored the lowest. Readability analysis indicated advanced text complexity. Senior ophthalmologists consistently provided lower ratings than junior clinicians did, highlighting expertise-related differences in perceived educational value. Sora Turbo can generate clinically useful anterior segment images for educational purposes, particularly for pathologies with prominent morphological features. This first systematic evaluation in ophthalmology demonstrates the promise of AI-generated atlases as scalable teaching resources for early-stage trainees while emphasizing the need for expert validation and ethical oversight before integration into formal curricula.

**Supplementary Information:**

The online version contains supplementary material available at 10.1038/s41598-025-27020-x.

## Introduction

The recent rise of text-to-image (T2I) generative artificial intelligence (AI) models has sparked widespread excitement^1^. These T2I tools translate textual descriptions into corresponding visual outputs. They typically employ deep neural networks, such as diffusion models or generative adversarial networks (GANs), trained on vast image-text datasets, which first encode the text into a semantic representation and then iteratively decode it into a novel image^2^. As the capabilities of T2I tools continue to be explored, they hold significant in the medical domain, such as in the areas of popularization, promotion, and diagnosis^2–4^. Recently, interest in applying T2I generative AI to medical education has increased^5,6^.

Several T2I models (such as Midjourney, DALL-E, Stable Diffusion and Gemini) are currently available and demonstrate utility in medical education^5^; however, a lack of anatomical precision and fidelity prevents these systems from satisfying the requirements of medical education^5^. For example, an evaluation of DALL-E 3 depictions of congenital heart disease revealed a high prevalence of anatomical inaccuracies (81%), incorrect annotations (85%), and content unusable for educational purposes (78%)^7^. Sora Turbo, an advanced text-to-video model developed by OpenAI, has recently expanded its capabilities to include T2I generation^8^. Unlike traditional T2I tools such as DALL-E, Sora Turbo generates images by leveraging its video frame generation approach, resulting in photorealistic visuals, naturalistic details, and high spatial fidelity. Therefore, Sora’s T2I capability may hold distinct potential for medical education.

Ophthalmic education needs to provide learners with more visual cues to enrich their clinical experience. Compared with other multimodal ophthalmic imaging modalities, slit-lamp anterior segment photography serves as the foundational cornerstone and represents the initial and primary focus for physicians in clinical diagnosis and management. Mastering the features of anterior segment photography in ocular diseases is an essential milestone in the professional development of every ophthalmologist. However, existing learning resources such as textbooks, journal articles, direct clinical observation, and online materials suffer from limitations, including restricted access, fragmented availability, inconsistent quality, and copyright or privacy constraints^9–11^. T2I generative AI models may offer solutions to address these challenges.

In this study, Sora Turbo was used to generate slit-lamp anterior-segment images across common entities, and their quality and educational applicability were evaluated.

## Materials and methods

**Fig. 1:**
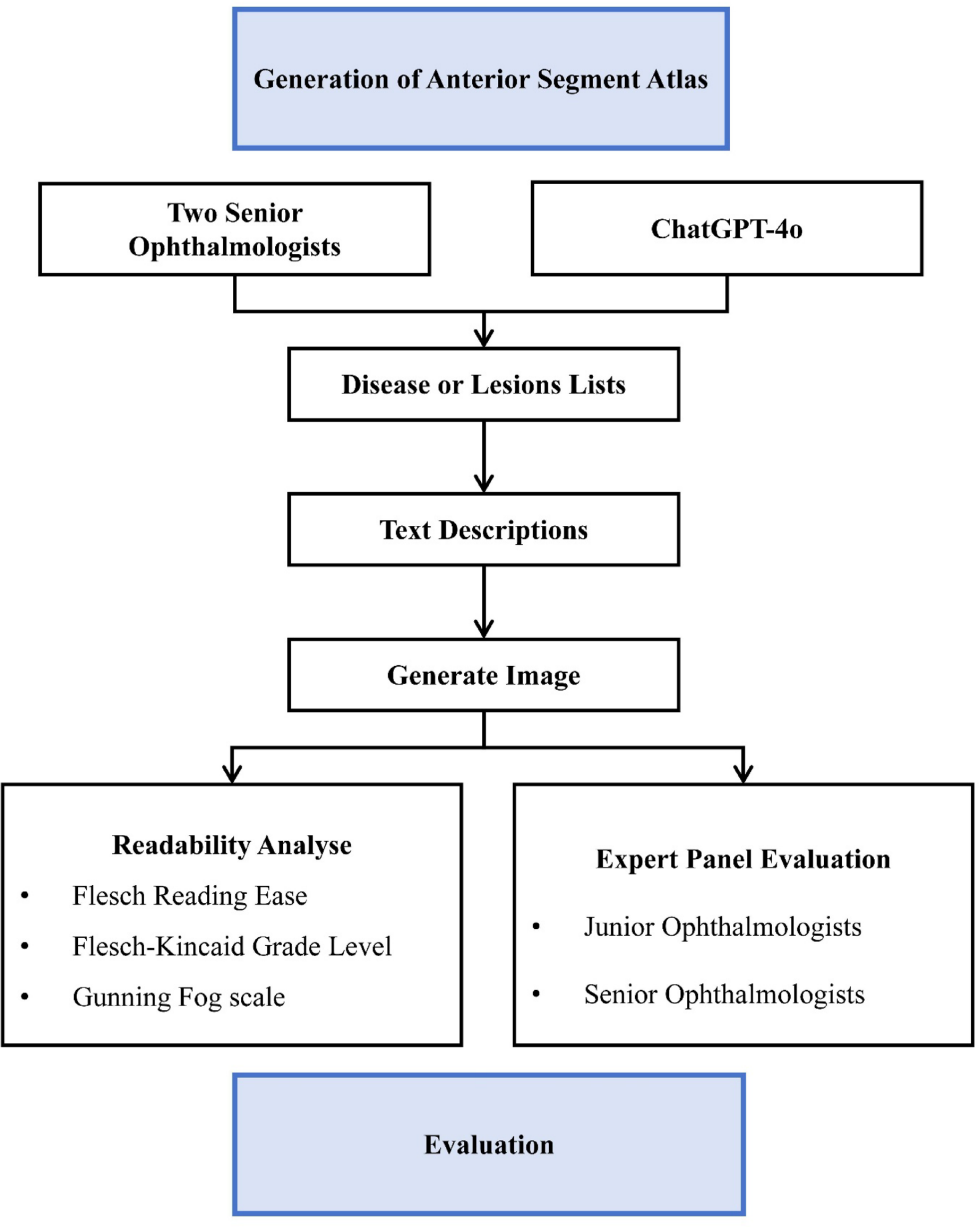
Flow chart.

Given that this study did not involve human subjects, human participants, protected health information (PHI), or patient data and relied exclusively on publicly accessible artificial intelligence tools for image generation, informed consent was not needed, and institutional ethics committee review and approval were not applicable (Fig. 1).

### Generation of the anterior segment atlas

Two senior ophthalmologists independently compiled lists of common ocular diseases or lesions of the anterior segment on the basis of their clinical experience. In parallel, a list of common diseases or lesions was generated using the GPT-4o (OpenAI, Inc., San Francisco, California, United States, July 14, 2025), guided by the prompts “*Please help list common ocular diseases or lesions of the anterior segment*”. Finally, the research team consolidated all three lists into the final, standardized version after a group discussion. The final inclusion criteria focused on three main factors: teaching importance (high frequency or exam relevance), anatomical coverage (ensuring that all major categories, such as the cornea, iris, and lens, were included), and graded difficulty (ranging from obvious, high-contrast signs to subtle findings).

For each anterior-segment disease or lesion, GPT-4o produced text descriptions conditioned on a structured prompt schema; a representative template was “*Give me a description of an ocular anterior segment photograph of XX*”. The Sora T2I model (OpenAI, Inc., San Francisco, California, United States, July 16, 2025) generated four high-resolution images per disease or lesion according to standard prompt specifications (*“Please generate an ocular anterior segment photograph of ‘XX’ for ophthalmic education. The characteristic features are ‘XX’.”*). All the images were generated in default mode (no preset), and per image was output at 1536 × 1024 pixels (PNG, aspect ratio 3:2). No manual retouching or postprocessing was performed. Four ocular anterior segment images paired with their corresponding textual descriptions for each disease or lesion collectively constitute the Anterior-Segment Atlas.

### Readability analysis

The Readability Analyzer (https://datayze.com/readability-analyzer) was used to assess the readability of the descriptive texts. For each descriptive text, the Flesch Reading Ease (FRE), Flesch‒Kincaid Grade Level (FKGL) and the Gunning Fog scale (GFS) were calculated. FRE is an algorithm that calculates text accessibility on the basis of average sentence length and syllable count per word^12^. FRE quantifies text simplicity on a 0–100 scale, where higher scores indicate easier comprehension^12^. Both the FKGL and the GFS are used to measure the years of education required for text comprehension^13,14^. Technically, FKGL and FRE share computational variables, whereas GFS differentially weights complex words in its readability assessment^13^.

### Anterior-segment photograph evaluation

An expert panel comprising junior (work experience < 10 years) and senior ophthalmologists (work experience ≥ 10 years) was convened to evaluate the anterior-segment atlas across five dimensions: (1) text accuracy (whether the accompanying textual descriptions used accurate medical terminology, were clearly expressed, logically structured, and objectively reflected the pathological features depicted in the images); (2) image reliability (whether the anterior-segment images presented clinically realistic representations, meeting standards sufficient for teaching use); (3) recognizability (whether the disease or pathological features could be reliably identified from the images alone, without reliance on supplemental information); (4) educational value (whether the combination of images and text provided practical value in ophthalmology teaching or self-study, enhancing disease recognition capabilities and deepening clinical understanding); and (5) generation stability (whether the four generated images per case demonstrated uniform, stable, and high-quality characteristics). All dimensions were evaluated using a 5-point Likert scale, where a score of 1 indicated the poorest evaluation and a score of 5 indicated the highest evaluation.

### Statistical analysis

The statistical analyses were conducted with SPSS software (IBM SPSS Statistics for Windows, Version 25.0.0; IBM, Armonk, New York, USA). Normality was assessed with the Shapiro–Wilk test; owing to nonnormality, continuous variables are summarized as medians (Q1–Q3), and categorical variables are summarized as frequencies (%). For between-group comparisons (junior vs. senior), scores were first aggregated at the entity level (within-group median per entity and dimension) and then compared using two-sided Mann–Whitney U tests with Benjamini–Hochberg false discovery rate (FDR) adjustment across the five dimensions; effect sizes (Cliff’s δ) were reported. Interrater agreement was quantified using Kendall’s W with tie correction, computed for the overall panel (n = 20) and within each seniority subgroup (n = 10); significance was evaluated via the χ^2^ approximation. A two-sided *p* < 0.05 was considered to indicate statistical significance.

## Results

The atlas incorporates 40 categories of anterior segment conditions, including normal images along with various pathologies, the latter of which covers 6 conjunctival diseases/pathologies, 8 lacrimal apparatus and eyelid diseases/pathologies, 8 corneal diseases/pathologies, 4 lens diseases/pathologies, 4 iris diseases/pathologies, and 9 unclassified diseases/pathologies. (Supplement Table 1).

**Table 1:** Readability analysis for textual descriptions of the anterior segment photography.

Diseases or lesions	FRE	GFS	FKGL
Normal	42.71	13.8	10.41
Conjunctivitis	29.2	15.95	12.56
Pterygium	38.53	12.14	11.08
Pinguecula	34.02	14.11	11.4
Conjunctival Papilloma	31.97	15.44	12.12
Conjunctival Melanoma	37.5	14.32	11.35
Subconjunctival Hemorrhage	34.03	14.95	11.65
Trichiasis	17.28	17.43	14.37
Ectropion	32.68	15.76	12.21
Entropion	34.74	16.01	11.5
Corneal ulcer	27.72	17.11	13.49
Band keratopathy	7.74	18.54	15.76
Corneal scar	38.48	13	11
Punctate Epithelial Keratopathy	31.11	13.64	11.84
Persistent Pupillary Membrane	47.9	14.27	9.95
Arcus senilis	29.61	15.64	12.43
Hordeolum	40.18	15	10.76
Chalazion	48.51	11.86	9.07
Posterior synechia	49.97	11.81	9.48
Cataract	29.06	18.25	12.9
Posterior capsular Opacificatio	20.41	18.76	14.25
Anterior capsular Contraction Syndrome	36.42	15.51	11.22
Implanted Intraocular lens	28.21	18.51	12.87
Hyphema	29.16	17.15	13.13
Hypopyon	8.77	20.92	16.26
Corneal Neovascularization	16.22	16.91	15.18
Acute dacryocystitis	40.1	14.26	11.05
Wearing a soft contact lens	46.4	12.89	10.02
Corneal suture after cataract surgery	18.38	19.33	14.42
Corneal edema	30.95	15.58	12.17
A history of radial keratotomy	51	13.23	9.91
A history of LASIK surgery	36.7	16.02	12.07
A history of SMILE surgery	46.52	15.59	10.43
A history of Penetrating keratoplasty	33.21	16.02	12.15
orbital cellulitis	32.31	16.81	11.77
Iridodialysis	33.41	15.07	12.01
A history of laser peripheral Iridotomy	37.35	16.14	11.92
Iris neovascularization	18.16	17.7	15.16
Ptosis	34.39	15.35	11.98
Corneal foreign body	35.18	15.13	12.37

FRE, Flesch reading ease, FKGL, Flesch-Kincaid grade level, GFS, The gunning fog scale.

Readability analysis revealed FRE scores ranging from 7.74 (band keratopathy) to 51 (a history of radial keratotomy), with FKGL values between 9.07 (chalazion) and 16.26 (hypopyon) and GFS scores ranging from 11.81 (posterior synechia) to 20.92 (hypopyon) (Table 1). Collectively, the readability metrics suggest that the textual descriptions are pitched at an advanced reading level appropriate for professional audiences.

Anonymized information on the evaluators is provided in Supplement Table 2. Across all the raters, agreement was statistically significant for all five dimensions, indicating low-to-moderate agreement overall. In the junior and senior subgroups, agreement remained significant with a similar pattern: image-related dimensions showed relatively higher agreement than text accuracy did. (Table 2).

**Table 2:** Inter-rater agreement (Kendall’s W) for overall and seniority subgroups.

Group	Dimension	Disease (N)	Rater (N)	Kendall’s *W*	*p*
Overall	Text accuracy	40	20	0.113	< 0.001
	Image reliability	40	20	0.359	< 0.001
	Recognizability	40	20	0.296	< 0.001
	Educational value	40	20	0.302	< 0.001
	Generation stability	40	20	0.257	< 0.001
Junior	Text accuracy	40	10	0.16	0.010
	Image reliability	40	10	0.38	< 0.001
	Recognizability	40	10	0.308	< 0.001
	Educational value	40	10	0.317	< 0.001
	Generation stability	40	10	0.285	< 0.001
Senior	Text accuracy	40	10	0.157	0.013
	Image reliability	40	10	0.415	< 0.001
	Recognizability	40	10	0.376	< 0.001
	Educational value	40	10	0.357	< 0.001
	Generation stability	40	10	0.302	< 0.001

**Fig. 2:**
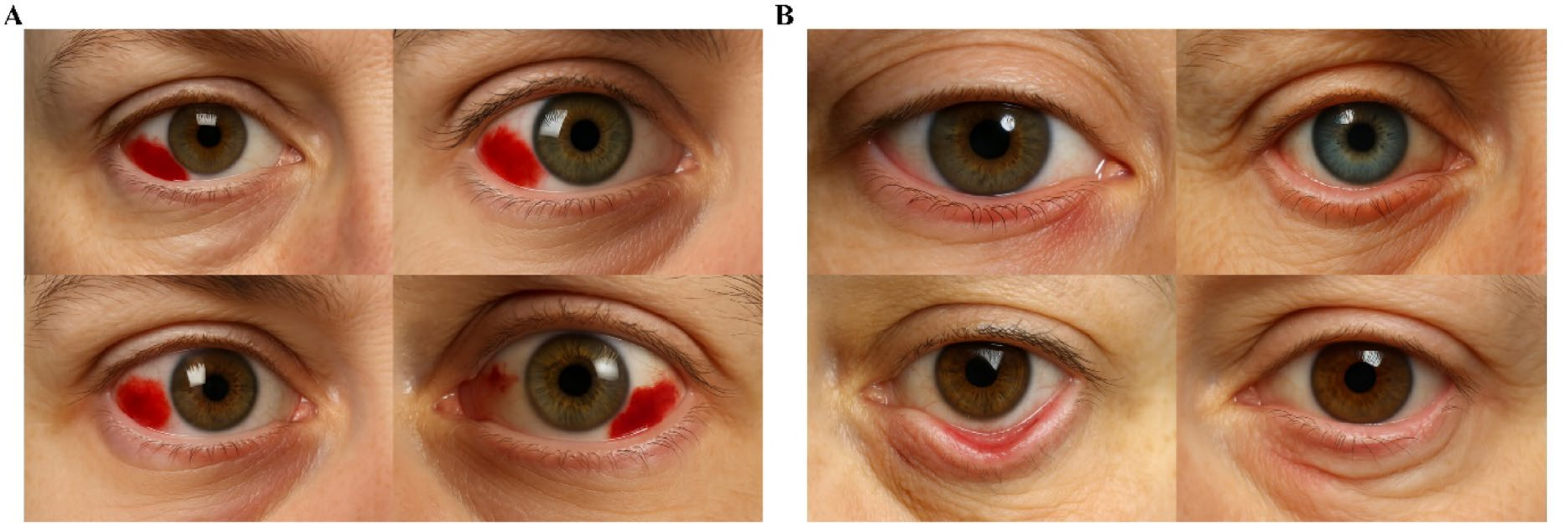
Examples of AI-generated anterior segment images. (**A**) One of the images with the highest total score (subconjunctival haemorrhage), (**B**) one of the images with the lowest total score (entropion).

Ten junior ophthalmologists and ten senior ophthalmologists were asked to assess the quality of the anterior-segment atlas. Table 3 presents the aggregated dimension-specific and composite scores for each image in the anterior segment atlas, which are based on assessments from all 20 ophthalmologists. All the images achieved relatively high text accuracy scores and generation stability scores (scores ≥ 3). However, lower scores (scores < 3) were observed for image reliability (entropion, corneal foreign body, hyphema, iridodialysis, a history of laser peripheral iridotomy), recognizability (entropion), and educational value (entropion, corneal foreign body). With respect to overall quality, only entropion and corneal foreign bodies exhibited poor image quality (total score ≤ 15), with entropion images receiving the lowest total score of 12.5 points. Five images (corneal neovascularization, cataract, subconjunctival haemorrhage, ptosis and normal anterior segment) achieved a maximum of 25/25 (Fig. 2).

**Table 3:** The result of anterior segment photograph evaluation.

Diseases or lesions	Text accuracy	Image reliability	Recognizability	Educational value	Generation stability	Total score
Normal	5 (4–5)	5 (4.25–5)	5 (5–5)	5 (4–5)	5 (5–5)	25 (23–25)
Conjunctivitis	4 (3.25–5)	4 (3–5)	5 (3.25–5)	5 (3–5)	5 (3.25–5)	22 (16.25–24.75)
Pterygium	4.5 (2–5)	4 (2–5)	5 (4–5)	4.5 (2.25–5)	4 (3–5)	21.5 (14–25)
Pinguecula	5 (3.25–5)	4 (3–5)	5 (4–5)	5 (4–5)	5 (3.25–5)	24 (20–25)
Conjunctival Papilloma	5 (4–5)	4 (3.25–5)	5 (4–5)	4.5 (4–5)	4.5 (3.25–5)	22 (19–25)
Conjunctival Melanoma	4.5 (4–5)	4 (3–5)	5 (4–5)	4.5 (3.25–5)	4.5 (3.25–5)	23 (16.75–24.75)
Subconjunctival Hemorrhage	5 (5–5)	5 (4–5)	5 (4–5)	5 (4.25–5)	5 (4.25–5)	25 (21.5–25)
Trichiasis	4 (4–5)	3.5 (3–4.75)	4 (2.25–5)	4 (3–5)	4 (3–4.75)	20 (15–23)
Ectropion	5 (4–5)	4 (3.25–5)	4 (3–5)	5 (4–5)	4 (4–5)	23 (18.25–25)
Entropion	4 (3–5)	2 (1–3)	1.5 (1–3)	2 (1–3)	3 (1–3.75)	12.5 (8.25–16.5)
Corneal ulcer	5 (4–5)	4.5 (3.25–5)	5 (4–5)	5 (3.25–5)	4.5 (4–5)	23.5 (20–25)
Band keratopathy	5 (4–5)	3.5 (2–4.75)	4 (3–5)	3.5 (2–5)	4.5 (3.25–5)	21 (14.25–23.75)
Corneal scar	5 (4–5)	4.5 (2.25–5)	5 (3–5)	5 (3–5)	5 (3–5)	24 (15.25–25)
Punctate Epithelial Keratopathy	5 (4–5)	4 (2–5)	5 (3.25–5)	4 (2.25–5)	5 (3.25–5)	23.5 (14.25–25)
Persistent Pupillary Membrane	5 (4–5)	4 (3–5)	4 (3–5)	4 (3–5)	4 (3.25–5)	20.5 (15.5–25)
Arcus senilis	5 (4–5)	4 (3–5)	4 (4–5)	4 (3–5)	4.5 (3.25–5)	22 (18.25–24)
Hordeolum	5 (4–5)	4.5 (3–5)	5 (4–5)	4.5 (3–5)	5 (4–5)	23 (18–25)
Chalazion	5 (4–5)	5 (3–5)	5 (3.25–5)	5 (3–5)	5 (4–5)	24.5 (17.25–25)
Posterior synechia	5 (4–5)	4 (3–5)	5 (3–5)	4.5 (3–5)	4.5 (3–5)	22.5 (15.25–25)
Cataract	5 (4.25–5)	5 (4–5)	5 (4–5)	5 (4–5)	5 (4–5)	25 (20.25–25)
Posterior capsular Opacificatio	5 (4–5)	4 (3–5)	4 (3–5)	4 (2.25–5)	5 (4–5)	22 (15.5–25)
Anterior capsular Contraction Syndrome	5 (4–5)	4 (2.25–4)	4 (3–4)	4 (2–4)	4 (3–5)	20 (15–22)
Implanted Intraocular lens	5 (4.25–5)	4.5 (4–5)	5 (4–5)	5 (4–5)	5 (4–5)	24 (20–25)
Hyphema	4.5 (3–5)	2 (1–3)	3 (2–4)	3 (1–4)	3.5 (2–4.75)	16.5 (10–19.75)
Hypopyon	5 (4–5)	4 (4–5)	5 (4–5)	5 (4–5)	5 (4–5)	23 (20.25–25)
Corneal Neovascularization	5 (4–5)	5 (3.25–5)	5 (4–5)	5 (3.25–5)	5 (4–5)	25 (18.75–25)
Acute dacryocystitis	5 (3.25–5)	3 (1.25–4)	4 (2–5)	4 (2–5)	4 (3–5)	19.5 (11.25–24)
Wearing a soft contact lens	5 (4–5)	4 (3–5)	4 (3–5)	3 (3–5)	4 (3–5)	19.5 (16.25–24.75)
Corneal suture after cataract surgery	4 (3–5)	3 (1.25–4)	3.5 (2–4.75)	3 (2–4)	3 (3–4.75)	16.5 (13–22)
Corneal edema	5 (4–5)	4 (4–5)	5 (4–5)	5 (4–5)	5 (4–5)	24 (20–25)
A history of radial keratotomy	5 (5–5)	5 (4–5)	5 (4–5)	5 (4–5)	5 (4–5)	24.5 (21.5–25)
A history of LASIK surgery	5 (4–5)	4.5 (3–5)	4.5 (3.25–5)	4.5 (3.25–5)	5 (3.25–5)	23 (16.75–25)
A history of SMILE surgery	5 (4–5)	5 (3.25–5)	5 (4–5)	5 (4–5)	5 (4–5)	24.5 (18.5–25)
A history of Penetrating keratoplasty	4.5 (4–5)	4 (3–5)	4.5 (4–5)	4.5 (4–5)	4.5 (3.25–5)	22.5 (19.25–24.75)
orbital cellulitis	5 (4–5)	4 (3–5)	4 (3–5)	4 (3–5)	4 (3–5)	21 (15–25)
Iridodialysis	4.5 (3.25–5)	2 (1–4.75)	3.5 (1.25–5)	3.5 (1.25–5)	3.5 (1.25–5)	16.5 (10–24.75)
A history of laser peripheral Iridotomy	4.5 (3.25–5)	2.5 (1–4)	3 (1–4.75)	3 (1–4.75)	3.5 (1–5)	16 (8.25–22.5)
Iris neovascularization	5 (4–5)	4 (2.25–5)	4.5 (3–5)	4.5 (2.25–5)	4.5 (3–5)	21.5 (15.25–25)
Ptosis	5 (5–5)	5 (4–5)	5 (4.25–5)	5 (4.25–5)	5 (5–5)	25 (22.5–25)
Corneal foreign body	4 (3–5)	2 (1–3)	3 (1.25–4.75)	2.5 (1.25–4.75)	3 (1–4.75)	15 (10–20.75)

**Fig. 3:**
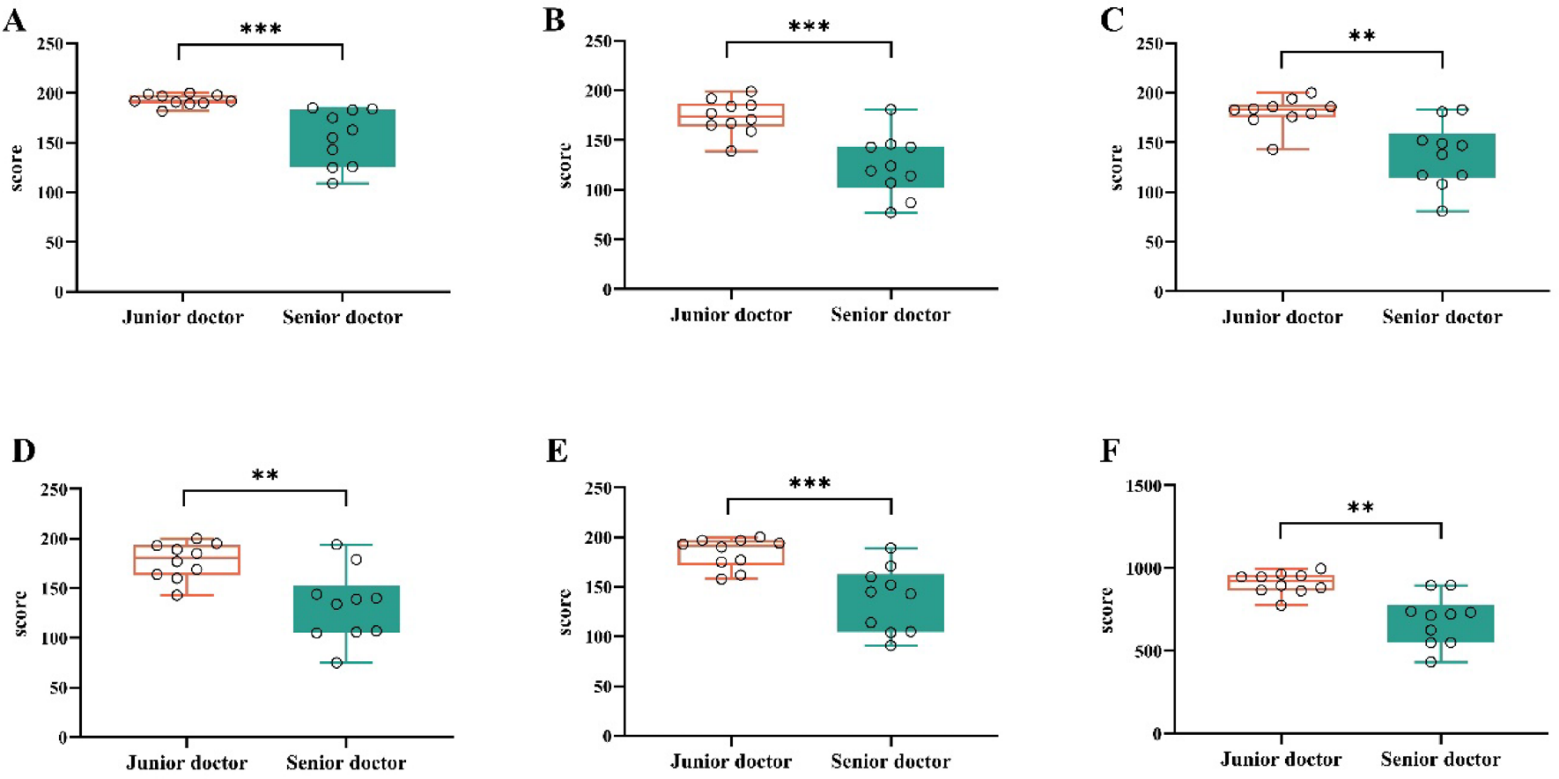
Distribution of the evaluation scores between the two groups of clinicians. (**A**) Text accuracy, (**B**) image reliability, (**C**) recognizability, (**D**) educational value, (**E**) generation stability, (**F**) total score.

We also evaluated differences in anterior segment image quality scores across the evaluator subgroups. Two-sided Mann–Whitney U tests with BH–FDR adjustment across the five dimensions revealed that all five dimensions remained significant after FDR correction (*p* < 0.001), with junior raters assigning higher scores than senior raters did. Effect sizes (Cliff’s δ) were large (approximately 0.80–0.88), indicating a robust and consistent difference in rating tendencies (Fig. 3 and Table 4). These findings indicate that senior clinicians apply stricter quality thresholds, whereas junior evaluators are more accepting of the synthetic atlas, suggesting particular value for early-stage training.

**Table 4:** Junior vs. Senior comparison.

Dimension	Junior (median)	Senior (median)	U	*p*	Cliff’s δ	*p*
Text accuracy	5	4	1457	< 0.001	0.875	< 0.001
Image reliability	5	3	1438	< 0.001	0.837	< 0.001
Recognizability	5	4	1469.5	< 0.001	0.798	< 0.001
Educational value	5	3.5	1500	< 0.001	0.821	< 0.001
Generation stability	5	4	1442	< 0.001	0.802	< 0.001

## Discussion

The rapid advancement of T2I generative models has opened new avenues for medical education, particularly in visually oriented specialties such as ophthalmology. Previous studies have explored the utility of T2I tools such as DALL-E and Midjourney in generating medical imagery, although they often highlight limitations in terms of anatomical accuracy and educational suitability^4,15^. In contrast, Sora Turbo, with its video-based image generation framework, offers enhanced photorealism and spatial fidelity, suggesting potential advantages for medical applications. This study represents the first systematic evaluation in ophthalmology of Sora Turbo’s capability to generate slit-lamp anterior segment images and assess their quality and educational value through expert review.

Our results reveal notable variations in image quality across different anterior segment entities. Conditions with well-defined and gross morphological features, such as cataracts, subconjunctival haemorrhage and ptosis, received consistently high scores across all dimensions. This finding indicates that Sora Turbo excels in rendering visually distinct and clinically recognizable pathologies. In contrast, entities requiring fine anatomical detail or dynamic contextual understanding—such as entropion, corneal foreign body, hyphema and iridodialysis—scored lower for reliability, recognizability and educational value. Our results are consistent with those of the study by Temsah et al., which evaluated DALL-E 3 to illustrate congenital heart disease (CHD)^7^. They reported a high prevalence of anatomical inaccuracies (80.8% of the images were rated as incorrect or fabricated), particularly for complex cardiac anomalies. Moreover, a comparative analysis of existing T2I models, such as Midjourney, Stable Diffusion, and DALL-E 3, in generating anatomy illustrations similarly highlighted significant limitations, such as inaccurate anatomical details and incomplete understanding of complex structures^5,16^. These findings suggest that while T2I models are highly capable of generating photorealistic images, their performance may be constrained by the complexity and subtlety of specific ophthalmic structures.

To further contextualize these findings, we compared our results to the performance of T2I models in other visually driven medical fields, specifically dermatology and radiology. Similar to our observations in ophthalmology, studies in dermatology have revealed significant concerns regarding the diagnostic accuracy and ethical readiness of AI-generated content. An evaluation of T2I models for common dermatological conditions revealed that the majority of the generated images severely underrepresented skin of colour (89.8% depicted light skin) and demonstrated poor overall diagnostic accuracy (maximum 22.5% across platforms)^17^. Moreover, expert scrutiny of skin cancer images confirmed that although DALL-E could capture some features, models such as Midjourney and BlueWillow often generated exaggerated, fictionalized elements deemed unsuitable for formal clinical education. In radiology, the discussion focuses on the potential for AI to generate highly realistic synthetic cardiac CT images, which can aid in creating diverse teaching cases and overcoming data privacy concerns^18^. However, this capacity simultaneously underscores ethical risk, challenging the integrity and trustworthiness of the data used for both research and education. Collectively, this cross-specialty comparison emphasizes that the successful integration of T2I technology into medical curricula requires a concerted effort to address pervasive issues of anatomical accuracy, algorithmic bias, and ethical oversight, regardless of the medical subspecialty.

With respect to readability assessment, quantitative readability metrics were incorporated into the evaluation system of this study, thereby establishing a relationship between visual content quality and textual cognitive load for better alignment with clinical applications. According to the U.S. Department of Health and Human Services (USDHHS), online information should be written at a 7th to 8th grade reading level, which aligns with the average American reading ability^19^. Our results indicate that a lower FRE score, together with higher FKGL and GFS, suggests that the generated ophthalmic atlases are more suitable for professionals, such as clinicians or medical trainees, than for public health education.

Senior ophthalmologists consistently assigned significantly lower scores across multiple evaluation dimensions than junior clinicians did. This discrepancy likely stems from the senior evaluators’ greater clinical experience and more rigorous diagnostic criteria, particularly regarding anatomical precision. These findings align with those of medical imaging studies, which have demonstrated that expertise is associated with more efficient visual search patterns and prolonged fixations on critical diagnostic regions, allowing experts to more readily identify subtle inaccuracies that may elude novices^20^. In contrast, junior clinicians who are still acquiring and developing ophthalmic diagnostic competencies may view AI-generated images as sufficiently accurate and pedagogically valuable for supplementary learning. These findings underscore the need to adapt educational materials to the trainee’s level of expertise, thereby positioning AI-generated atlases as particularly valuable for early-stage ophthalmology training, whereas advanced learners may require higher-fidelity or clinically validated images.

In this study, interrater agreement among 20 experts for five dimensions across 40 entities was evaluated using Kendall’s W. Agreement was statistically significant across all dimensions and subgroups (junior/senior) but exhibited a low-to-moderate magnitude. This magnitude is plausible because of three factors: (1) the inherent subjectivity of text accuracy (which requires heterogeneous internal thresholds for lexical precision and implied factual consistency), (2) the attenuating effect of frequent ties generated by the 5-point Likert scale on the W coefficient, and (3) heterogeneity in rater background and training^21^. Crucially, agreement was consistently greater for image-related dimensions. From an educational perspective, this low-to-moderate agreement does not undermine utility, as final metrics rely on aggregated scores (mean/median). The stable, significant agreement in image-related dimensions supports their use as alignable instructional objectives and reliable assessment indicators, while lower-agreement dimensions (text accuracy) can be improved through the implementation of standardized scoring rubrics and prerating calibration exercises^22^.

Beyond addressing the current need for high-quality static ophthalmic images, the generated atlas holds significant potential to enhance future educational modalities, particularly in clinical simulations and remote learning^23,24^. As T2I technology advances, these synthetic atlases can evolve from static images to dynamic, AI-augmented learning environments^25^. This evolution would enable trainees to interact with simulated virtual patients, where the AI model rapidly generates variations in disease presentations on the basis of user-defined parameters (e.g., severity, patient demographics, and comorbidities). Such dynamic simulation is critical for developing clinical decision-making skills and diagnostic flexibility. Furthermore, for remote learning and geographically disparate educational programs, these AI-enhanced atlases offer an equitable solution by providing unlimited, standardized, and high-quality simulated clinical cases, effectively reducing the disparities in learning resources across different institutions^26^.

Despite the promise of AI-generated content, integrating T2I models such as Sora Turbo mandates considering ethical risks, specifically training bias and Deepfake misuse^27^. Generative AI models are trained on vast, unfiltered internet datasets, inevitably encoding societal biases. In ophthalmology, this limitation translates to the potential for the atlas to propagate diagnostic biases related to demographics or underrepresented lesion presentations^27^. Such bias could subtly distort visual features, potentially affecting diagnostic pattern recognition when trainees encounter real-world diversity. Furthermore, the model’s capacity to generate highly photorealistic images presents the ethical challenge of deepfakes^28,29^. The ease of synthesizing convincing medical images necessitates strict governance to prevent their misinterpretation as real patient data. The required rigorous validation by clinical experts and ongoing supervision by interdisciplinary ethics committees, as emphasized in our conclusion, remains essential to vet content for clinical accuracy and the absence of harmful bias^30^.

Several limitations should be considered. First, the expert panel included 20 ophthalmologists from a limited set of institutions, which may introduce selection bias and constrain generalizability. Moreover, agreement was estimated on ordinal 5-point ratings where ties attenuate W; subgroup precision is limited by a fixed number of raters. We prioritized Kendall’s W (agreement in rankings) over the ICC given the scale and study aim. Future multicentre work with larger rater pools and standardized rubrics may yield higher agreement and narrower uncertainty. Finally, the evaluation was restricted to curated, static images; dynamic light or tear phenomena, depth cues, and three-dimensional relationships were not assessed, nor were the effects of calibration.

## Conclusion

In this study, Sora Turbo-generated slit-lamp images of the anterior segment were evaluated. Although the model produced realistic images for gross pathologies, it struggled with fine anatomical structures. Senior ophthalmologists rated images more critically than junior ophthalmologists did, reflecting the influence of clinical expertise on evaluation. AI-generated image atlases show particular promise for early-stage ophthalmology training. Future implementation must include expert validation and ethical oversight to ensure educational safety and accuracy.

## Supplementary Information

Supplementary Material 1

## Data Availability

The data that support the findings of this study are available from the corresponding author, Xuanqiao Lin, upon reasonable request.
