# Supplementary material for: Assessing the quality and educational applicability of AI-generated anterior segment images in ophthalmology
Source: Sci Rep. 2025 Nov 28;15:42778. doi: 10.1038/s41598-025-27020-x (PMC12663094; doi:10.1038/s41598-025-27020-x)
Supplement: Supplementary file 1 — Supplementary Material 1 [file 41598_2025_27020_MOESM1_ESM.docx]

**Supplemental Digital Content**

Supplement Table 1: Textual descriptions and screening details of the anterior segment images.

Supplement Table 2: Per-rater profile

| **Supplement Table 1 Textual descriptions and screening details of the anterior segment photographs** | |
| --- | --- |
| Diseases or lesions | Textual descriptions |
| Normal | This high-resolution external eye photograph shows the eye of a healthy adult individual in primary gaze. The eyelids are symmetrical and of normal position, with no evidence of swelling, ptosis, or entropion/ectropion. The conjunctiva appears white and clear, with no signs of injection or lesions. The cornea is transparent and smooth, without vascularization, scarring, or opacities. The sclera is white, and the limbus is sharply defined. The iris is clearly visible with a normal, round pupil centered in the visual axis. There is no discharge, redness, or abnormal pigmentation. Overall, the eye appears normal and free of pathology. |
| Conjunctivitis | A high-resolution, realistic color photograph of a human eye with acute conjunctivitis. The image should show the eye in primary gaze, with clear visualization of the upper and lower palpebral conjunctiva and bulbar conjunctiva. The conjunctiva appears diffusely hyperemic with prominent blood vessel dilation and redness, especially in the inferior fornix. There is mild to moderate chemosis (conjunctival swelling) and watery discharge near the medial canthus. The cornea remains clear, and the pupil is round and reactive. The eyelashes and eyelids appear normal. No text, labels, or annotations should appear anywhere in the image. |
| Pterygium | A high-resolution, realistic color photograph of a human eye with pterygium. The image should show the eye in primary gaze, with clear visualization of the nasal bulbar conjunctiva and cornea. A triangular-shaped, fleshy, pinkish lesion extends from the nasal conjunctiva onto the cornea, with its base at the limbus and its apex pointing toward the central cornea. The surface of the lesion is slightly elevated with visible superficial blood vessels. The surrounding conjunctiva may appear mildly hyperemic. The cornea outside the lesion remains clear. Eyelids, lashes, and sclera appear normal. No text, labels, or annotations should appear anywhere in the image. |
| Pinguecula | This high-resolution external eye photograph shows the nasal bulbar conjunctiva of a patient with a pinguecula. A small, slightly elevated, yellowish lesion is visible adjacent to the limbus, located in the interpalpebral fissure zone. The lesion has a smooth surface and well-defined borders. It does not invade the cornea and is separated from the limbus by a clear margin. The surrounding conjunctiva may appear mildly hyperemic. The cornea, iris, and pupil are normal. The eyelids and eyelashes appear unremarkable. These features are consistent with a benign conjunctival degeneration known as pinguecula. |
| Conjunctival Papilloma | This high-resolution external eye photograph shows a patient with a conjunctival tumor located on the bulbar conjunctiva. The lesion appears as a well-defined, elevated mass with a fleshy, irregular surface. It may be pink, gray-white, or lightly pigmented, with visible feeder vessels on or around the lesion. The mass is typically located near the limbus but may extend onto the cornea or into the fornix. The surrounding conjunctiva may show localized hyperemia. The cornea, iris, and pupil appear uninvolved. The eyelids and eyelashes are normal. These features are suggestive of a conjunctival neoplasm such as ocular surface squamous neoplasia (OSSN), conjunctival papilloma, or melanoma. |
| Conjunctival Melanoma | A high-resolution, realistic color photograph of a human eye with conjunctival melanoma. The image should show the eye in primary gaze, with a pigmented lesion located on the bulbar conjunctiva near the limbus or extending toward the fornix. The lesion appears dark brown to black, irregular in shape, slightly elevated, and has poorly defined or asymmetric borders. Prominent feeder vessels may be present over or surrounding the lesion. The lesion may extend onto the corneal surface, but the central cornea remains clear. Surrounding conjunctiva may show localized injection or thickening. Eyelids and eyelashes appear normal. No text, labels, or annotations should appear anywhere in the image. |
| Subconjunctival Hemorrhage | A high-resolution, realistic color photograph of a human eye with a subconjunctival hemorrhage. The image should show the eye in primary gaze, clearly displaying the bulbar conjunctiva. A well-demarcated, bright red or dark red patch is visible beneath the transparent conjunctiva, typically located in the temporal or nasal scleral region. The hemorrhage has smooth borders and does not extend onto the cornea. There is no associated swelling, discharge, or conjunctival elevation. The pupil, iris, cornea, and eyelids appear normal. The surrounding conjunctiva remains clear outside the hemorrhagic area. No text, labels, or annotations should appear anywhere in the image. |
| Trichiasis | This high-resolution external eye photograph shows a case of trichiasis. Several eyelashes are misdirected and growing posteriorly toward the ocular surface, instead of projecting outward. The affected lashes are typically seen along the lower or upper eyelid margin and may contact the bulbar conjunctiva or cornea. Mild conjunctival injection is present, and the corneal surface may show subtle punctate staining or localized epithelial irregularity in areas of lash contact. The eyelid margin appears otherwise structurally intact, without entropion. These features are consistent with trichiasis. |
| Ectropion | This high-resolution external eye photograph shows a patient with lower eyelid ectropion. The lower eyelid margin is turned outward, away from the globe, resulting in poor apposition of the lid to the ocular surface. The palpebral conjunctiva is exposed and visible, often appearing red and thickened due to chronic irritation. The lower punctum is everted and displaced from its normal position, leading to impaired tear drainage and secondary epiphora (tearing). The eyelashes are directed downward or outward, and do not contact the ocular surface. The sclera, iris, and cornea appear unaffected. These features are consistent with involutional ectropion. |
| Entropion | This high-resolution external eye photograph shows a patient with entropion involving the lower eyelid. The eyelid margin is turned inward toward the globe, causing multiple eyelashes to contact or rub against the ocular surface. The inward rotation of the lid results in poor apposition of the lid margin to the globe. Several misdirected lashes are seen touching the bulbar conjunctiva or cornea. Mild conjunctival injection and signs of ocular surface irritation are visible. The upper eyelid appears normal. These findings are consistent with lower eyelid entropion. |
| Corneal ulcer | This high-resolution external eye photograph shows a patient with a corneal ulcer. The cornea has a localized, grayish-white or yellowish opacity, often with an irregular surface and poorly defined borders. The ulcer is typically central or paracentral, and may appear slightly elevated or depressed relative to the surrounding clear cornea. There is marked conjunctival injection, especially ciliary (limbal) injection, giving the eye a red, inflamed appearance. In some cases, a small hypopyon may be visible in the inferior anterior chamber. The eyelids may appear swollen or mildly ptotic, and the patient may exhibit excessive tearing or photophobia. |
| Band keratopathy | This anterior segment OCT image shows band keratopathy as a hyperreflective, linear deposition within the anterior corneal layers. The deposits are localized to the Bowman’s layer and subepithelial space, forming a continuous or segmented band across the interpalpebral region. The corneal epithelium overlying the deposits may appear thinned or irregular. The stromal and posterior corneal surfaces remain intact. The hyperreflective band is more pronounced nasally and temporally, corresponding to calcium salt accumulation. |
| Corneal scar | A high-resolution, realistic color photograph of a human eye in primary gaze, showing a corneal scar. A localized, whitish or grayish opacity is visible in the cornea. The lesion has irregular but well-defined borders and appears as a stable, non-elevated stromal opacity. The corneal surface over the scar is smooth, with no active ulceration or epithelial defect. The surrounding cornea is clear, and the iris and pupil are visible through the transparent areas. There is no conjunctival injection, discharge, or neovascularization. The eyelids and lashes appear normal. No text, labels, or annotations should appear in the image. |
| Punctate Epithelial Keratopathy | A high-resolution, realistic photograph of a human eye under cobalt blue light illumination after fluorescein staining, showing punctate epithelial keratopathy (PEK). Multiple small, bright green dot-like stains are scattered across the corneal surface, especially in the central and inferior regions. The staining pattern is non-confluent and highlights superficial epithelial damage. The iris and pupil are visible in the background. The surrounding conjunctiva may show mild injection. The eyelids and eyelashes appear normal. The image should resemble a slit-lamp fluorescein photo. No text, labels, or annotations should appear in the image. |
| Persistent Pupillary Membrane | A high-resolution, realistic color photograph of a human eye in primary gaze, showing persistent pupillary membrane (PPM). Several fine, thread-like strands are visible crossing the pupil, extending from one part of the iris to another, or from the iris to the anterior lens capsule. The strands are translucent or lightly pigmented and appear suspended in the aqueous. The pupil is round and centered, with no distortion. The iris and cornea appear normal. There are no signs of inflammation, posterior synechiae, or lens opacity. No text, labels, or annotations should appear in the image. |
| Arcus senilis | This high-resolution external eye photograph shows a patient with arcus senilis. A grayish-white, ring-like opacity is visible along the peripheral cornea, near the limbus. The arc appears bilaterally symmetric and typically spares a clear zone between the arc and the limbus (the lucid interval of Vogt). The central cornea remains transparent. The ring is more prominent superiorly and inferiorly, gradually forming a complete circle with age. The iris, pupil, and conjunctiva appear normal. This appearance is characteristic of lipid deposition in the peripheral corneal stroma, commonly seen in elderly individuals. |
| Hordeolum | This high-resolution external eye photograph shows a patient with an acute hordeolum affecting the upper eyelid. There is a localized, red, tender, and swollen area near the eyelid margin. A small yellowish point or pustule may be visible at the center of the swelling, indicating a developing abscess. The overlying skin appears tense and inflamed. The eyelashes adjacent to the lesion are normal. The conjunctiva may be mildly injected due to secondary irritation. The cornea, iris, and sclera are unaffected. These findings are consistent with an external hordeolum (stye), typically caused by infection of a Zeis or Moll gland. |
| Chalazion | A high-resolution, realistic color photograph of a human eye with a chalazion on the upper eyelid. A firm, non-tender, round nodule is visible under the skin of the upper eyelid, causing mild elevation without redness. The skin appears smooth and unbroken. There is no central pustule or signs of acute inflammation. The eyelashes and eyelid margin are normal. The conjunctiva and cornea are unaffected. The iris and sclera are clearly visible. No text, labels, or annotations should appear in the image. |
| Posterior synechia | A high-resolution, realistic color photograph of a human eye in primary gaze, showing posterior synechia. The pupil is irregularly shaped, with one or more segments of the iris visibly adhered to the anterior surface of the lens. These adhesions distort the contour of the pupil, making it appear oval, notched, or asymmetrical. The iris is slightly pulled toward the lens at the points of adhesion. The iris texture and color are otherwise normal. The cornea and conjunctiva are clear. No text, labels, or annotations should appear in the image. |
| Cataract | This high-resolution external eye photograph shows a patient with a cataract following pharmacologic pupil dilation. The pupil is widely dilated, revealing the underlying crystalline lens. The lens appears cloudy and opacified, with varying degrees of whiteness or yellowing depending on the cataract type. In nuclear cataracts, the central lens is yellow or brown; in cortical cataracts, radial spoke-like opacities may be visible; and in mature cataracts, the entire lens appears diffusely white or milky. The iris is fully visible, and the cornea remains clear. The conjunctiva and sclera appear normal. These findings are consistent with lens opacity visualized through a dilated pupil. |
| Posterior capsular Opacificatio | This high-resolution external eye photograph shows a pseudophakic eye with posterior capsular opacification following cataract surgery. After pharmacologic dilation, the pupil is widely open, allowing a clear view of the intraocular lens (IOL) and the posterior capsule. A central, grayish-white, hazy area is visible behind the IOL optic, representing opacification of the posterior capsule. The opacity may appear fibrotic or granular and partially obscures the red reflex. The IOL itself is well-centered, and both its anterior and posterior surfaces are visible. The iris is normal, and the cornea is clear. These findings are consistent with posterior capsular opacification, a common delayed complication after IOL implantation. |
| Anterior capsular Contraction Syndrome | A high-resolution, realistic color photograph of a human eye after intraocular lens (IOL) implantation, showing severe anterior capsular contraction syndrome. The pupil is fully dilated, but the anterior capsular opening is markedly constricted and fibrotic. A thick, white, circular fibrotic band overlaps the IOL optic, leaving only a small, central pinhole-like clear zone. The fibrotic capsule is dense, shrunken, and contracts symmetrically around the optic. The IOL appears slightly decentered. The iris is visible and evenly pigmented, and the cornea is clear. No inflammation is present. No text, labels, or annotations should appear in the image. |
| Implanted Intraocular lens | This high-resolution external eye photograph shows a pseudophakic eye after pharmacologic pupil dilation. The pupil is widely dilated, allowing a clear view of the implanted intraocular lens (IOL). The IOL optic is round, well-centered, and transparent, located in the posterior chamber behind the iris. Both the anterior and posterior surfaces of the IOL are smooth and visible through the dilated pupil. The iris is evenly colored and fully visible. The cornea appears clear, and there are no signs of inflammation, synechiae, or capsular opacification. These findings represent a normal postoperative appearance after cataract surgery with successful IOL implantation. |
| Hyphema | This high-resolution external eye photograph shows a patient with hyphema, characterized by visible blood accumulation in the anterior chamber of the eye. A horizontal reddish fluid level is clearly seen in the inferior part of the anterior chamber, settling due to gravity. The extent of the hyphema may vary depending on the severity, from a small layered meniscus (microhyphema) to a total hyphema that obscures the entire pupil and iris. The cornea is clear, allowing direct visualization of the blood layer. The iris and pupil may be partially or completely obscured. The conjunctiva may appear injected, especially in traumatic cases. These findings are consistent with a traumatic or spontaneous hyphema. |
| Hypopyon | This high-resolution external eye photograph shows a patient with hypopyon, characterized by a distinct yellowish-white fluid level in the inferior portion of the anterior chamber. The hypopyon appears as a horizontal meniscus composed of layered leukocytes and inflammatory cells, often sharply demarcated from the clear aqueous above. The cornea is typically clear, allowing visualization of the fluid level, although corneal edema may be present in severe inflammation. The iris and pupil are partially obscured. The conjunctiva may show significant hyperemia or ciliary injection, indicating underlying anterior segment inflammation. These findings are consistent with anterior uveitis or endophthalmitis with hypopyon formation. |
| Corneal Neovascularization | This high-resolution external eye photograph shows corneal neovascularization involving the peripheral and paracentral cornea. Fine to moderately sized blood vessels are seen extending from the limbus into the normally avascular corneal stroma. These vessels appear as red, branching lines that traverse the clear corneal tissue, often oriented radially from the conjunctiva. The affected corneal area may appear slightly hazy or whitish due to associated stromal inflammation or scarring. The central cornea may remain relatively clear, depending on severity. The conjunctiva is mildly injected, and the pupil and iris remain visible through the cornea. These findings are consistent with superficial and/or deep corneal neovascularization, commonly seen in contact lens overwear, chronic inflammation, or limbal stem cell deficiency. |
| Acute dacryocystitis | This high-resolution external eye photograph shows a patient with acute dacryocystitis. There is marked swelling, redness, and tenderness over the lacrimal sac area, located inferomedial to the medial canthus of the eye. The overlying skin appears erythematous and tense, with a dome-shaped or crescent-shaped raised lesion in the region of the nasolacrimal sac. The swelling may extend toward the lower eyelid and nose. The conjunctiva is mildly injected, and epiphora (overflow of tears) may be present. In severe cases, a yellowish point may suggest abscess formation. The upper and lower eyelids may show reactive edema. These findings are consistent with acute inflammation of the lacrimal sac. |
| Wearing a soft contact lens | This high-resolution external eye photograph shows a healthy eye wearing a soft contact lens. The lens is transparent and covers the central cornea, conforming closely to its curvature. Its edges are faintly visible as a subtle circular outline on the corneal surface, especially near the limbus. The cornea underneath appears clear, with no signs of irritation, redness, or vascularization. The conjunctiva and sclera are white and uninjected. The eyelids are in a normal position. The iris and pupil are clearly visible through the contact lens, indicating that the lens is well-centered and clean. These findings represent a normal contact lens–wearing eye. |
| Corneal suture after cataract surgery | This high-resolution external eye photograph shows a postoperative pseudophakic eye following cataract surgery. A single fine monofilament suture is visible at the temporal-superior limbal region. The suture passes vertically through the corneoscleral junction, perpendicular to the limbus, indicating the location of the clear corneal or corneoscleral incision. The surrounding cornea is transparent and shows no signs of edema or infiltration. The conjunctiva near the incision site may show mild hyperemia. The iris and a well-centered intraocular lens (IOL) optic are visible through the clear cornea. This represents a typical early postoperative appearance after small-incision cataract surgery with a single suture closure. |
| Corneal edema | This high-resolution external eye photograph demonstrates corneal edema in a patient with anterior segment pathology. The normally transparent cornea appears diffusely hazy and swollen, with a grayish or bluish tint. The stromal edema causes loss of corneal clarity and a dull reflection of light. Fine folds in Descemet's membrane (Descemet's folds) may be visible as subtle linear striae, particularly in the central or inferior cornea. The iris and pupil are partially obscured behind the edematous cornea. In severe cases, the corneal epithelium may appear microcystic or bullous. The conjunctiva may be mildly injected. These findings are consistent with moderate to severe corneal edema. |
| A history of radial keratotomy | This high-resolution external eye photograph shows the eye of a patient with a history of radial keratotomy (RK). Multiple thin, linear, radial corneal scars extend from the peripheral cornea toward the central optical zone, forming a starburst-like pattern. The incisions are well-healed and appear as white or gray lines in the corneal stroma, typically numbering 8 to 16 cuts. The central cornea is relatively clear but may show mild haze depending on the severity. The iris and pupil are visible through the central clear zone. The sclera and conjunctiva appear normal, with no signs of injection. These findings are classic for a post-RK eye. |
| A history of LASIK surgery | This high-resolution external eye photograph shows the eye of a patient who has undergone LASIK surgery. The cornea appears clear and smooth, with a faint horizontal or circular demarcation line representing the edge of the corneal flap, typically located in the superior or superotemporal region. The flap interface may show subtle light reflection differences but without significant haze or scarring. The limbus, sclera, and conjunctiva appear normal, with no signs of injection. The iris and pupil are clearly visible through the transparent cornea. These findings are consistent with a well-healed postoperative LASIK eye. |
| A history of SMILE surgery | This high-resolution external eye photograph shows the eye of a patient after undergoing Small Incision Lenticule Extraction (SMILE) refractive surgery. The cornea appears clear, smooth, and free of haze or visible scars. Unlike LASIK, there is no visible corneal flap or flap edge. In some cases, a faint, small peripheral incision—usually temporal or superior temporal—may be seen near the limbus, representing the entry site for lenticule extraction. The pupil and iris are clearly visible through the transparent cornea. The conjunctiva and sclera appear normal, with no signs of injection. These findings are consistent with a well-healed post-SMILE eye. |
| A history of Penetrating keratoplasty | This high-resolution external eye photograph shows a patient who has undergone penetrating keratoplasty. A central circular corneal graft is visible, sharply demarcated from the surrounding host cornea. Multiple interrupted or continuous 10-0 nylon sutures are present at the graft-host junction, appearing as fine black threads arranged circumferentially. The graft appears clear, with minimal haze, and the iris and pupil are visible through the transplant. The surrounding conjunctiva and sclera appear normal, with no significant injection. These findings represent a well-healed post-PKP eye. |
| orbital cellulitis | This high-resolution external eye photograph shows a patient with orbital cellulitis affecting the left eye. The periorbital tissues are markedly swollen and erythematous, with diffuse edema involving the upper and lower eyelids. The eyelid skin appears tense, shiny, and red. The eye is partially or fully closed due to swelling. There is visible proptosis, and the globe appears pushed forward. The conjunctiva is chemotic, and the sclera is injected. Extraocular movements may appear restricted or painful (not visible in the photo). These findings are consistent with acute orbital cellulitis, typically secondary to sinus infection or trauma. |
| Iridodialysis | This high-resolution photograph shows a patient with iridodialysis, a separation of the iris root from its attachment at the ciliary body. A crescent-shaped peripheral defect is visible at the iris base, typically in the inferotemporal quadrant. Through the defect, the peripheral retina or zonular fibers may be visible, creating a second pseudo-pupil appearance. The primary pupil may appear irregular or slightly displaced. The rest of the iris appears intact. The condition is often caused by blunt ocular trauma or intraocular surgery. The cornea is clear, and the lens and anterior chamber are visible. |
| A history of laser peripheral Iridotomy | This high-resolution external eye photograph shows the eye of a patient after undergoing laser peripheral iridotomy (LPI). A small, well-defined round or oval opening is visible at the peripheral iris, most commonly in the superior quadrant between 10 and 2 o’clock positions. The iridotomy appears as a dark red to black spot through which the posterior chamber is visible. The surrounding iris tissue is intact, and the cornea remains clear. The pupil is round and centrally positioned. There is no visible inflammation or hemorrhage, indicating a well-healed postoperative state. These findings are consistent with a completed and patent LPI, commonly performed to prevent or treat angle-closure glaucoma. |
| Iris neovascularization | This high-resolution slit-lamp photograph shows a close-up view of the anterior segment of an eye with iris neovascularization (rubeosis iridis). Fine, delicate, and irregular blood vessels are seen on the anterior surface of the iris stroma, most prominently around the pupillary margin and extending toward the mid-periphery. These neovessels are tortuous and disorganized, lacking the normal radial pattern of iris vasculature. Some vessels may bridge across the iris surface or form loops. In advanced cases, neovascularization may also extend into the anterior chamber angle, though not visible in this view. The pupil may appear slightly irregular, and the anterior chamber is otherwise quiet. These findings are characteristic of rubeosis iridis, often associated with proliferative diabetic retinopathy or central retinal vein occlusion. |
| Ptosis | This high-resolution external eye photograph shows a patient with unilateral ptosis of the left upper eyelid. The upper eyelid margin is abnormally low, covering a portion of the pupil and resting significantly below the superior limbus. The palpebral fissure is visibly narrowed compared to the contralateral eye. The levator function may appear reduced, though not directly assessable in a static image. The lower eyelid position is normal. The globe is in primary gaze, and the cornea, iris, and pupil are otherwise normal and symmetric between the two eyes. These findings are consistent with moderate left upper eyelid ptosis. |
| Corneal foreign body | This high-resolution external eye photograph shows a small metallic foreign body embedded in the peripheral cornea, located in the temporal quadrant away from the central visual axis. The foreign body appears as a dark, sharply defined speck with mild surrounding stromal haze. There is localized conjunctival injection adjacent to the lesion, and a subtle corneal epithelial reaction can be seen around the impact site. The central cornea remains clear, with no involvement of the visual axis. The pupil and iris are visible and unaffected. These findings are consistent with a superficial, non-central corneal foreign body. |

| **Supplement Table 2 Per-rater profile** | | | |
| --- | --- | --- | --- |
| **Rater** | **Years of experience** | **Subspecialty** | **Country** |
| R01 | 3 | Cornea | China |
| R02 | 2 | Glaucoma | China |
| R03 | 4 | Refractive Surgery | China |
| R04 | 4 | Oculoplastics | Korea |
| R05 | 3 | Comprehensive | Japan |
| R06 | 7 | Cornea | China |
| R07 | 9 | Cataract | India |
| R08 | 4 | Cataract | Malaysia |
| R09 | 6 | Comprehensive | India |
| R10 | 6 | Cornea | China |
| R11 | 12 | Refractive Surgery | China |
| R12 | 14 | Glaucoma | China |
| R13 | 14 | Cataract | Japan |
| R14 | 19 | Oculoplastics | China |
| R15 | 11 | Comprehensive | Korea |
| R16 | 12 | Cataract | China |
| R17 | 13 | Glaucoma | Singapore |
| R18 | 15 | Cataract | China |
| R19 | 18 | Refractive Surgery | China |
| R20 | 20 | Cornea | China |
| Years of experience = years after MD/after ophthalmology training | | | |
